# *VdP5CDH* is involved in melanin formation, stress resistance and play a regulatory role in virulence of *Verticillium dahliae*

**DOI:** 10.3389/fmicb.2024.1429755

**Published:** 2024-07-24

**Authors:** Wanqing Sun, Lihong Zhao, Jinglong Zhou, Hongjie Feng, Yalin Zhang, Zili Feng, Heqin Zhu, Feng Wei

**Affiliations:** ^1^Zhengzhou Research Base, State Key Laboratory of Cotton Bio-breeding and Integrated Utilization, School of Agricultural Sciences, Zhengzhou University, Zhengzhou, China; ^2^State Key Laboratory of Cotton Bio-breeding and Integrated Utilization, Institute of Cotton Research of Chinese Academy of Agricultural Sciences, Anyang, Henan, China; ^3^Western Agricultural Research Center, Chinese Academy of Agricultural Sciences, Changji, Xinjiang, China

**Keywords:** *V. dahliae*, carboxylate dehydrogenase, melanin, nutrient utilization, pathogenicity

## Abstract

**Introduction:**

*Verticillium dahliae*, a soil-borne fungal pathogen, can cause cotton Verticillium wilt. In this study, VdP5CDH, the member of the ALDH_F4-17 family of carboxylate dehydrogenases, was identified in the genome of *V. dahliae* and investigated function in regulating virulence by generating gene deletion mutants and complementary mutants.

**Methods:**

Homologous recombination method was used to construct mutants, transcriptome sequencing revealed gene-related metabolic pathways, and disease degree of cotton was observed through pathogen infection experiments.

**Results:**

The conidial surface of *VdP5CDH* deletion strains was dented and shriveled, and the number of conidial spores increased. Compared with the wild-type (WT), the mycelial diameter of deletion mutants increased by 10.59%-11.16%, the mycelial growth showed irregular branching patterns, and misaligned arrangement. Although capable of penetrating cellophane, deletion mutants were unable to produce melanin. *VdP5CDH* was mainly associated with glucose metabolism, nitrogen metabolism, ABC transporter activity as well as various amino acid metabolic processes. After gene knockout, raffinose and pectin were used as the main carbon sources to promote the growth of strains and the growth rate of deletion strains in the medium containing raffinose was higher than that of WT. Consequently, the deletion mutant strains decreased utilization efficiency with which they utilized various nitrogen sources. The deletion mutants maintain responsiveness to osmotic stress and oxidative stress stimuli. Additionally, compared to WT strains, the deletion mutant strains exhibited differences in culture temperature tolerance, UV exposure response, and fungicide sensitivity. After cotton was infected with deletion strains conidial suspension, its disease index increased dramatically, while it gradually decreased after spraying with 2 mM glutamate in batches. With the increase of spraying times, the effect was more significant, and the disease index decreased by 18.95%-19.66% at 26 dpi.

**Discussion:**

These results indicated that *VdP5CDH* regulates the pathogenicity of fungi and controls mycelia growth, melanin formation, conidia morphology, abiotic stress resistance, and the expression of infecting structure-related genes.

## 1 Introduction

*Verticillium dahliae* is a soil-borne pathogenic fungus that can cause wilt disease in a variety of plants, including numerous agricultural crops, and woody plants (Fradin and Thomma, 2006). *V. dahliae* exhibits long-term survival in the soil by forming microsclerotia, which serve as the primary infection source and display exceptional resistance to extreme temperatures and other adverse environmental circumstances (Fan et al., 2017). Under the stimulation of the secretion from the root system of host plant, microsclerotium germinates into the mycelium, enabling the infectious structures entry into plant roots. Subsequently, fungal colonization occurs within xylem vessels followed by systemic expansion through transpiration, which causes leaf blight and yellowing, root vascular bundle browning, and plant diseases index rising (Klosterman et al., 2009; Lo Presti et al., 2015). So far, the regulation mechanism of gene in virulence is still unclear. There is an urgent need to analyze the function of genes involved in molecular mechanisms.

Genes may play a role by regulating amino acid metabolism pathways (Deuschle et al., 2004; Sahoo et al., 2004). The initial and rate-limiting stage in the oxidative decomposition of proline is the oxidation of proline to generate pyrroline-5-carboxylic acid (P5C), which is catalyzed by the FAD-dependent proline dehydrogenase (ProDH) (Servet et al., 2012). In contrast, pyrroline-5-carboxylate dehydrogenase (P5CDH) is a NAD-dependent enzyme responsible for converting P5C to glutamate (Cabassa-Hourton et al., 2016). It mainly exists in mitochondria and is encoded by universally expressed genes (Klosterman et al., 2009). ProDH binds to the inner membrane of mitochondria and its active site oriented towards the mitochondrial matrix, in which P5CDH also plays a crucial role in metabolic reactions (Elthon and Stewart, 1981). Exogenous proline can induce the expression of P5CDH (Deuschle et al., 2001). Failure to metabolize accumulated proline promptly may lead to damage or even cell death (Hellmann et al., 2000).

The biological morphology of pathogens is also regulated by related genes. Ubiquitin ligase (E3) *VdBre1* plays a pivotal role in various biological processes, including mycelial growth, conidia production, lipid metabolism and the secondary metabolism (Wang et al., 2021). In recent years, several crucial genes related to micronucleus and melanin formation have been identified. The mitogen-activated protein VMK1 regulates micronucleus growth and preserves cell wall integrity (Luo et al., 2016). For instance, the fungal specific transcription factor encoding gene *Vdpf* facilitates melanotic microsclerotium formation and conidial generation (Luo et al., 2016).

Nutrient uptake is regulated by genes and influences growth and metabolism of pathogens. Trace elements such as iron and a multitude of elements including carbon and nitrogen are essential nutrients in the growth and reproduction processes of *V. dahliae* (Ramachandra et al., 2014). Nitrogen can limit the colonization and basic metabolic processes of most organisms (Gaudinier et al., 2018). Pathogens must obtain sufficient nitrogen from their plant hosts during infection. Beyond just assisting in the nutrition supply, nitrogen metabolism serves as a crucial regulatory signal in the development of fungi (Deng et al., 2015). Pathogenic fungi have evolved complex mechanisms for acquiring nitrogen, and there are classified into dominant nitrogen sources and secondary nitrogen sources, such as ammonium and glutamine (Ries et al., 2018). When the dominant nitrogen source is absent, enzymes and permeable membranes are expressed at higher concentrations, then the second nitrogen source supplies metabolic energy, and the nitrogen-related synthesis pathway turns to the decomposition process (Tudzynski, 2014). Through construction of targeted gene deletion mutants, expression analysis, biochemical index determination, and pathogenicity experiments, it has been confirmed that the transcription factor *VdAtf1* regulates pathogenesis by controlling nitrogen nutrient utilization and nitrite resistance (Fan et al., 2017).

The gene affected the normal growth of strains by changing stress resistance. Accumulation of penetrating and low-mass molecules and the synthesis of antioxidant compounds can stimulate the antioxidant enzyme system and enhance osmotic regulation (Fahad et al., 2017). *VdDpb4* and *VdIsw2* are involved in maintaining chromatin structure for nucleosome positioning and transcriptional regulation, including genes associated with DNA repair in response to ROS stress during plant infection. Among pathogens such as *Penicillium* and *C. anthracis*, NO inhibits fungal growth mainly by inducing the generation of reactive oxygen species, which can lead to severe oxidative damage (Wang and Higgins, 2005). Benzovindiflupyr is beneficial to the prevention and control of pathogenic fungus, however, frequent usage of the chemical may accelerate the development of fungicide resistance in pathogen populations, so it is crucial to investigate strain resistance to different drugs (Li et al., 2023). The deletion mutant of RNA binding protein (RBP) VdNop12 exhibits increased sensitivity to low-temperature stress (Zhang et al., 2020).

Regulatory factors stimulate metabolism and affect fungal virulence. The absence of *VdSsk1*, a regulatory factor related to the toxicity of *V. dahliae* and homologous to *S. cerevisiae*, significantly diminishes the virulence of this pathogenic fungus (Zheng et al., 2019). Glutamate is essential for plant growth and stress resistance. as well as for the biosynthesis of proline and other nitrogen-containing compounds (Okumoto et al., 2016). Amino acids can stimulate both primary and secondary metabolic processes. The exogenous application of glutamate has been shown to enhance photosynthetic activity and leaf function, improving the quality of leeks by reducing nitrate accumulation (Franzoni et al., 2021). Similar effects have also been observed in water lettuce (Haghighi, 2012).

Recently, *VdP5CDH* (*VDAG_03218*) was identified as a pathogenicity-associated gene through RNA-Seq analysis. In this study, significant alterations were observed in the mycelial micromorphology and conidial morphology of *VdP5CDH* deletion mutants, accompanied by inhibited melanin production. The efficiency of carbon and nitrogen nutrients utilization decreased, while the resistance to oxidation stress increased. Notably, experimental data demonstrated that the absence of *VdP5CDH* enhanced the virulence of *V. dahliae*, *VdP5CDH* negatively regulated the pathogenicity of fungus.

## 2 Materials and methods

### 2.1 Pathogenic strains, plant culture and experimental conditions

The strain Vd080 was selected as the WT, and different mutants were generated through homologous recombination. Conidial suspensions of all *V. dahliae* strains for experiments were stored long-term at −80°C in 40% glycerol. Unless otherwise specified, the pathogenic fungal strains were cultured in solid medium such as potato-dextrose agar (PDA), liquid medium such as Czapek-Dox medium, or potato-dextrose broth (PDB). Jimian11, a susceptible variety of *G. hirsutum*, was cultivated in the greenhouse at 25°C (8 h/16 h dark/light cycle). The *N. benthamiana* was also cultured under the same temperature conditions as Jimian11.

### 2.2 Construction of *VdP5CDH* mutants

Gene deletion and complementation transformants were generated via *Agrobacterium* -mediated transformation (ATMT) method. Using the Vd080 genomic DNA as template, the primers B303-*VdP5CDH*-UP-F/R and B303-*VdP5CDH*-DOWN-F/R were used to amplify the upstream fragment 1.2 kb and downstream fragment 1.2 kb of the *VdP5CDH* genome sequence, respectively. The corresponding hygromycin fragment was amplified using the primers B303-*VdP5CDH*-Hyg-F/R with the B303 vector as template. The primers used was listed in Supplementary Table S2.

Gene encoding sequence was replaced with a hygromycin resistance cassette via homologous recombination to generate gene deletion mutants. Verification of gene amplification was performed using three pairs of primers. The amplified fragments were ligated into the B303 vector. Subsequently, the plasmid containing the positive recombinant vectors (B303-*VdP5CDH*-UP/Hyg/DOWN) were transformed into *Agrobacterium* AGL-1 (Liu et al., 2013), and the deletion mutants were isolated and screened on PDA solid medium supplemented with 50 μg/mL hygromycin for PCR verification. The upstream fragment and *VdP5CDH* genomic DNA were amplified with primers pSULPH-*VdP5CDH* (+UP)-F/R, with the total length of the two fragments was 2.2 kb. The complementary mutant of *VdP5CDH* was successfully constructed by connecting the upstream gene fragment with the full length of *VdP5CDH* genome to the pSULPH-mut-RG#PB vector. The plasmid carrying the fusion products was transferred to AGL-1, followed by mixing it with the filtrate of the complementary strains in a certain proportion, and then evenly coating it onto a solid medium surface for further screening of complementary mutants on PDA medium supplemented with 100 μg /mL chlorimuron-ethyl. The primers used was listed in Supplementary Table S2.

### 2.3 Determination of *VdP5CDH* enzyme activity

Agar disk containing mycelium from the WT, deletion mutants (*ΔVdP5CDH*) and complementary mutants (*C-ΔVdP5CDH*) were incubated in PDB at 25°C with continuous shaking for 5 d, then the mycelial suspension was filtered with four layers of gauze to remove solid debris. The enzyme activity was evaluated using a double-antibody sandwich assay. The rate of dehydrogenation was quantified by monitoring the reduction and subsequent discoloration of the chromogenic indicator. Following the formation of the antibody–antigen–enzyme conjugate, the complex was subjected to a chromogenic reaction, and the absorbance was measured at 450 nm using a spectrophotometric microplate reader. For the expression of *VdP5CDH*, the prokaryotic vector pCold-TF-*VdP5CDH* was constructed by inserting the coding sequence (CDS) of *VdP5CDH* into the pCold-TF vector. The recombinant plasmid was transformed into *E. coli* (BL21), and cultures were grown at 37°C for 5 h until reaching the logarithmic phase of growth. Induction of recombinant protein expression was achieved by adding 50 μL 1 mM isopropyl β-D-1-thiogalactopyranoside (IPTG) to the culture, followed by incubation at 16°C and 160 rpm for 12 h. The recombinant protein was subsequently purified using the His-tag Protein Purification Kit (Beyotime, Shanghai) and its enzymatic kinetics were analyzed using a microbial pyrrolin-5-carboxylate dehydrogenase (P5CDH) ELISA kit (MEIMIAN, Jiangsu). All experiments were conducted in triplicate to ensure reproducibility.

### 2.4 *VdP5CDH* induced cell death in *Nicotiana benthamiana*

The CDS fragment of the target gene was amplified from the Vd080 cDNA template and cloned into the PVX vector pGR107. Subsequently, the recombinant vector was transformed into the *A. tumefaciens* strain GV3101 (Gui et al., 2017). The bcl-2-associated X protein (BAX) and green fluorescent protein (GFP) served as positive and negative controls, respectively. *N. benthamiana* leaves were utilized as experimental material for transient gene expression analysis (Wang et al., 2022). All *Agrobacterium* solutions were cultured on a shaking incubator at 28°C for 2 d, followed by three rounds of suspension and adjustment to an optical density OD_600_ = 0.8. Then, the *N. benthamiana* leaves at 4 weeks of age were infiltrated with *Agrobacterium* infection solutions, and cultured in a greenhouse for 3 to 7 d to observe leaf lesion symptoms. The photographs were taken to document the results. The experiment was performed on 3 leaves from 3 different plants and repeated 3 times.

### 2.5 Observation of mycelial growth and conidia morphology

For the radial growth rate experiment, 5 μL conidial suspensions containing WT, *ΔVdP5CDH* and *C-ΔVdP5CDH* strains were inoculated at the center of PDA medium with a suspension concentration of 5 × 10^6^ cfu/mL, then the medium was air-dried and cultivated in a 25°C incubator for 14 d, after which the mycelial growth diameter was measured. The experiment was repeated three times. After culturing for 7 d, the agar disk containing mycelium were collected from the edge of the mycelia with an 8-mm-diameter borer and washed in 1 mL sterile water. The number of conidia in the sterile water was observed under a microscope (Tian et al., 2021). The microscopic morphology of high concentration conidia suspension was observed using a scanning electron microscope. After 7 d of cultivation, the aseptic cover glass was inserted into the growth edge of the mycelia and further cultured for an additional 3 d to observe the morphology of mycelia on the cover glass using fluorescence microscopy. The experiment was repeated three times.

### 2.6 Production of microsclerotium and melanin

The sterilized microporous filter membrane was positioned at the center of the basal modified medium (BMM) (0.2 g/L NaNO_3_, 0.52 g/L KCl, 0.52 g/L MgSO_4_·7H_2_O, 1.52 g/L KH_2_PO_4_, 3 mM thiamine, 0.1 mM biotin, 5 g/L glucose, and 15 g/L agar, pH = 7.6) (Hu et al., 2014). Conidial suspensions of WT, *ΔVdP5CDH* and *C-ΔVdP5CDH* strains, each with a concentration of 5 × 10^6^ cfu/mL were uniformly applied onto the microporous filter membrane, respectively. The BMM medium was incubated in a dark incubator at a temperature of 25°C for a duration of 40 d to observe and capture images of microsclerotia production. Each experiment was repeated in triplicate.

### 2.7 Penetration and microscopic morphology of mycelia

The 5 μL conidial suspensions of both WT and mutant strains, each with a concentration of 5 × 10^6^ cfu/mL were filtered, then sterile cellophane was positioned at the center of the PDA solid medium and the filtrate was titrated directly onto the sterile cellophane. The solid medium was incubated at 25°C for a duration of 3 d (Tian et al., 2021). Morphological characteristics of the mycelium on the cellophane were documented using photographic techniques. Under aseptic conditions, the cellophane film was removed and the plate was sealed to continue the incubation of the mycelia at 25°C for an additional 3 d. The cellophane covered with mycelia was cut into fixed-size rectangular strips and placed in a specimen chamber for observation under scanning electron microscopy (SEM). Each experimental iteration was conducted in triplicate.

### 2.8 RNA-seq and metabolic pathway analysis

Agar disk containing mycelium of WT and *ΔVdP5CDH* strains were inoculated in PDB liquid medium and cultured on a shaking table at 25°C for 5 d, then the mycelia were filtered using four layers of gauze and transferred to an alcohol-sterilized mortar. The mycelia were frozen under the action of liquid nitrogen and ground into fine powder, then stored into −80°C refrigerator. The resultant sample underwent a series of experimental procedures, including RNA extraction, library construction, transcriptome sequencing (Personalbio, Nanjing) and data analysis. Finally, KEGG and GO analyses were conducted to elucidate the relevant metabolic pathways.

### 2.9 Utilization effects of *ΔVdP5CDH* strains to multiple nutrients

Agar disk containing mycelium of the WT, *ΔVdP5CDH* and *C-ΔVdP5CDH* strains were inoculated into Czapek-Dox liquid medium and incubated at 25°C in a shaking incubator for 7 d, respectively. The filtered conidial suspensions were titrated onto W-S (water agar solid medium) supplemented with various carbon sources (10 g/L pectin, 10 g/L xylan, 10 g/L raffinose, 17 g/L starch and 30 g/L sucrose) (Qi et al., 2016). Subsequently, then the medium plates were incubated at 25°C for 14 d, with W-S medium without sucrose serving as the control. Colony morphology was documented through photography, and colony diameters were measured. The experiment was repeated in triplicate.

Agar disk of the WT and mutant strains were inoculated into Czapek-Dox liquid medium and cultured at 25°C for 7 d. Subsequently, the conidial suspensions were filtered and titrated onto the center of the Czapek-Dox solid medium supplemented with 10 mM different nitrogen sources (proline, glutamine, ammonium sulfate, nitrate, and asparagine), respectively. The medium was air-dried, sealed, and incubated at 25°C for 14 d. The Czapek-Dox medium without NaNO_3_ served as the control. Colony diameters were measured, and the colony morphology was documented (Wong et al., 2008). The experiment was conducted in triplicate.

### 2.10 Resistance of *ΔVdP5CDH* strains to different stresses

Abiotic stress agents such as 0.002% SDS, 0.02% Congo red, 1 M sorbitol, 1 M KCl and 1 M NaCl were individually supplemented into PDA solid medium, respectively (Yu et al., 2019). Subsequently, conidial suspensions of WT, *ΔVdP5CDH* and *C-ΔVdP5CDH* strains were titrated to the center of the medium and incubated at 25°C for 14 d. The growth diameter of each strain was measured and the colony morphology was documented through photography. The colony growth diameters of the WT strains cultured on the medium without any stress agent were used as the control. The experiment was conducted in triplicate.

The PDA was supplemented with fenpropidin (1 μg/mL), tebuconazole (0.1 μg/mL), spiroxamine (0.5 μg/mL), tridemorph (2 μg/mL), and triadimefon (20 μg/mL), respectively (Yu et al., 2019). The 10 μL conidia filtrates of WT, *ΔVdP5CDH* and *C-ΔVdP5CDH* strains were titrated onto the center of the plates, and the medium was incubated at 25°C for 14 d to observe changes in colony diameters under different pesticide stresses. The diameters of WT strains cultured on the medium without any pesticides were used as the control. This experiment was conducted in triplicate.

Sensitivity of different strains to cold (16°C), heat (37°C) and ultraviolet radiation stress was investigated. Conidial filtrates of WT and mutant strains were inoculated at the center of the PDA medium and then incubated at various temperatures (16°C, 25°C, 30°C, 37°C) for a duration of 14 d or treated with a 10 s pulse of 302 ηm ultraviolet radiation before culturing at 25°C for 14 d (Liu et al., 2017). The colony diameters of WT strains without culture temperature and UV radiation stress were regarded as control. The experiment was independently repeated three times.

The 5 mL conidial filtrate, with a concentration 5 × 10^6^ cfu/mL for both WT and mutant strains, was separately added to non-solidified PDA medium. The liquid medium was mixed and poured into a culture dish. A sterile circular filter paper with a diameter of 3 mm was placed at the center of each culture dish. Subsequently, 10 μL H_2_O_2_ solution with mass fractions of 15 and 30% were titrated onto the filter paper. The culture dishes were incubated at 25°C for 5 d, and the sizes of the inhibition zone were observed and measured. The WT strains treated under same culture conditions were used as the control. Each experiment was repeated three times.

### 2.11 Regulation of *VdP5CDH* on virulence

Cotton seeds of the disease susceptible variety Jimian11 were soaked for 12 h, then sown into paper cups filled with vermiculite. Subsequently, the cotton seedlings were cultivated in a greenhouse after being watered. Once the cotton seedlings grew two true leaves, the cotton roots were inoculated with the filtered conidial suspension (5 × 10^6^ cfu/mL) of WT, *ΔVdP5CDH* and *C-ΔVdP5CDH*. The disease index was calculated at 14 d, 20 d, and 26 d post-inoculation (Zhu et al., 2021). Three consecutive batches of cotton seedlings were planted, with multiple seedlings inoculated with the suspension of each strain to be tested.

The regulatory effect of *VdP5CDH* on glutamate production in fungi was explored through the detection of glutamate content. The WT, *ΔVdP5CDH*, and *C-ΔVdP5CDH* strains were cultured in Czapek-Dox liquid medium and incubated with agitation at 25°C for a duration of 5 d. The mycelium was isolated using four layers of gauze, followed by natural air-drying and pulverization into powder form. The powdered sample underwent acid hydrolysis by the addition of hydrochloric acid solution, facilitated by vortex mixing for thorough mixing and constant temperature hydrolysis. Subsequently, sodium hydroxide solution was introduced to the cooled hydrolysis mixture for neutralization. The supernatant was acquired via centrifugation, to which sodium bicarbonate solution and DNFB solution were added. This mixture was then subjected to a constant temperature water bath reaction in the absence of light. Finally, the resultant sample was prepared for analysis and subjected to instrumental testing.

To investigate the effect of glutamate spraying on pathogenicity, different concentrations of glutamate (2 mM, 4 mM, and 6 mM) were sprayed to cotton seedlings at various time points: 2 d before inoculation, and 3 d, 10 d, 16 d, and 20 d after inoculation. Cotton seedlings inoculated with WT conidial solution were subjected to each concentration of glutamate spray to assess its impact on the severity of leaf disease. Subsequently, a new batch of cotton seedlings was inoculated with the suspensions of Vd080, *ΔVdP5CDH* and *C-ΔVdP5CDH* strains, respectively. The concentration of glutamate that proved to be most beneficial for reducing disease was applied for further spraying experiment, while observing disease symptoms in plants infected with both WT and mutant strains. The experiment was repeated three times.

Examining the virulence effects of WT and mutant strains on the cotton seedling growth, the disease phenotypes of three batches of cotton plants were documented after 26 d of cultivation (Zhu et al., 2021). The disease index was calculated as follows: 100 × ∑ (number of diseased leaves at each level × representative value at each level)/(total number of leaves investigated × highest representative value). The degree of vascular tissue browning in the longitudinal section of the plant stems was recorded using the stereoscopic optical microscopy. Simultaneously, the cotton stems that had not been cut longitudinally were sectioned into consistent length branches. These branches were repeatedly cleaned and disinfected with sodium hypochlorite and 75% alcohol, then placed flat on PDA medium to isolate the fungus from the vascular bundle for subsequent growth (Zhao et al., 2016), followed by documentation.

At 26 dpi, the truncated cotton stems were pulverized into powder using a liquid nitrogen grinding method. The E.Z.N.A. High performance (HP) fungal DNA kit (Omega Bio-Tek, America) was used to extract plant total genomic DNA, and the DNA concentration was measured using the NanoDrop 2000. Subsequently, the DNA samples were utilized for real-time fluorescence quantitative PCR (qPCR) reaction. The ChamQ universal SYBR qPCR master mix (Vazyme, Nanjing) was used for quantitative analysis. The *V. dahliae* gene *β-tubulin* (GenBank registration number: DQ266153) was used to mark the amount of fungal colonization and the cotton gene *UBQ7* was used as an internal reference (Wen et al., 2023). Three independent replicates were performed to ensure reliability and reproducibility of the results. Primers was shown in the Supplementary Table S2.

### 2.12 Analysis of gene expression

Quantitative analysis was conducted on genes associated with the pathogenic process. The pathogenic genes associated with conidial production include *VdNLP1* (*VDAG_04701.1*), *VdPLP* (*VDAG_00942*) and *Vdpf* (*VDAG_08521.1*); the infection peg-related genes *VdNoxB* (*VDAG_09930*) and *VdPls1* (*VDAG_01769*); the transcriptional activator *VdCrz1* (*VDAG_03208*); follower protein related to the partial assembly of mycelia neck ring *VdSep5* (*VDAG_04382*); the appressorium structure-regulating gene *VdCSIN1* (*VDAG_05652*); Furthermore, two transcription factors, *Som1* (*VDAG_JR2_Chr1g09120a*) and *Vta3* (*VDAG_Chr1g07600a*), affect the adhesion, penetration and colonization of pathogens. Further quantitative analysis was performed on eight genes related to melanin production, including *Vayg1* (*VDAG_04954*), *VT4HR* (*VDAG_03665*), *VaflM* (*VDAG_00183*), *VdSCD* (*VDAG_03393*), *VAYG1* (*VDAG_04954*), *VT4HR* (*VDAG_03665*), *VDH1* (*VDAG_02273*), and *VdLAC* (*VDAG_00189*) (Hu et al., 2014). The primers used was listed in Supplementary Table S2.

Agar disk of the medium used to cultivate WT, *ΔVdP5CDH* and *C-ΔVdP5CDH* strains were transferred into Czapek-Dox liquid medium and cultured at 25°C for 7 d. The mycelia were collected by filtration with 4 layers of gauze and ground into powder using liquid nitrogen. Total fungal RNA was extracted using the Fungal Total RNA Isolation Kit (Sangon Biotech, Shanghai). Then the RNA was reverse transcribed into cDNA using HiScript II Q RT supermix for qPCR (genomic DNA [gDNA] wiper) (Vazyme, Nanjing) reagent kit. Using fungal cDNA as template, Reverse transcription-quantitative PCR (RT-qPCR) was conducted with SYBR green (Vazyme, Nanjing). The reaction steps of RT-qPCR experiment successively included an initial denaturation at 95°C for 30 s followed by a cycle process that repeated 40 times at 95°C for 3–10 s and 60°C for 10–30 s, and the collection of dissolution curve at 95°C for 15 s, 60°C for 60 s and 95°C for 15 s. Threshold cycle (2^-ΔΔCT^) method was used to calculate the relative gene expression. The *β-tubulin* (GenBank accession no. DQ266153) was used as the reference. The mean and standard error of gene expressions were estimated from three biological replicates.

### 2.13 Statistical analysis of data

The SPSS statistical software package (v22.0) was used to analyze the experimental data. One-way analysis of variance (ANOVA) was applied and followed by the student’s *t*-test to determine significant differences between treatments at *p* values of 0.05 or 0.001.

## 3 Results

### 3.1 Generation of *VdP5CDH* mutants

The genes involved in virulence differentiation processes were listed in Supplementary Table S1. Gene deletion mutants were generated using a homologous recombination strategy (Supplementary Figure S1A), and the deletion of *VdP5CDH* was confirmed through PCR analyses. Hygromycin fragment could be detected in the deletion mutants, while the target gene fragment of *VdP5CDH* was not present (Supplementary Figure S1B). The complete cDNA sequence of *VdP5CDH* was cloned from the highly pathogenic defoliating strain Vd080, and the pSULPH-*VdP5CDH* vector was constructed. Gene replacement was performed using the *Agrobacterium*-mediated transformation (ATMT) method. The upstream promoter region and coding sequence of *VdP5CDH* were reintroduced into gene deletion mutants. This complementation was further validated through PCR assays, and the hygromycin fragment was not present in the complementary mutants, but the target gene fragment of *VdP5CDH* could be detected (Supplementary Figure S1C).

### 3.2 Determination of VdP5CDH enzyme activity and its ability to induce cell death

**Figure 1:**
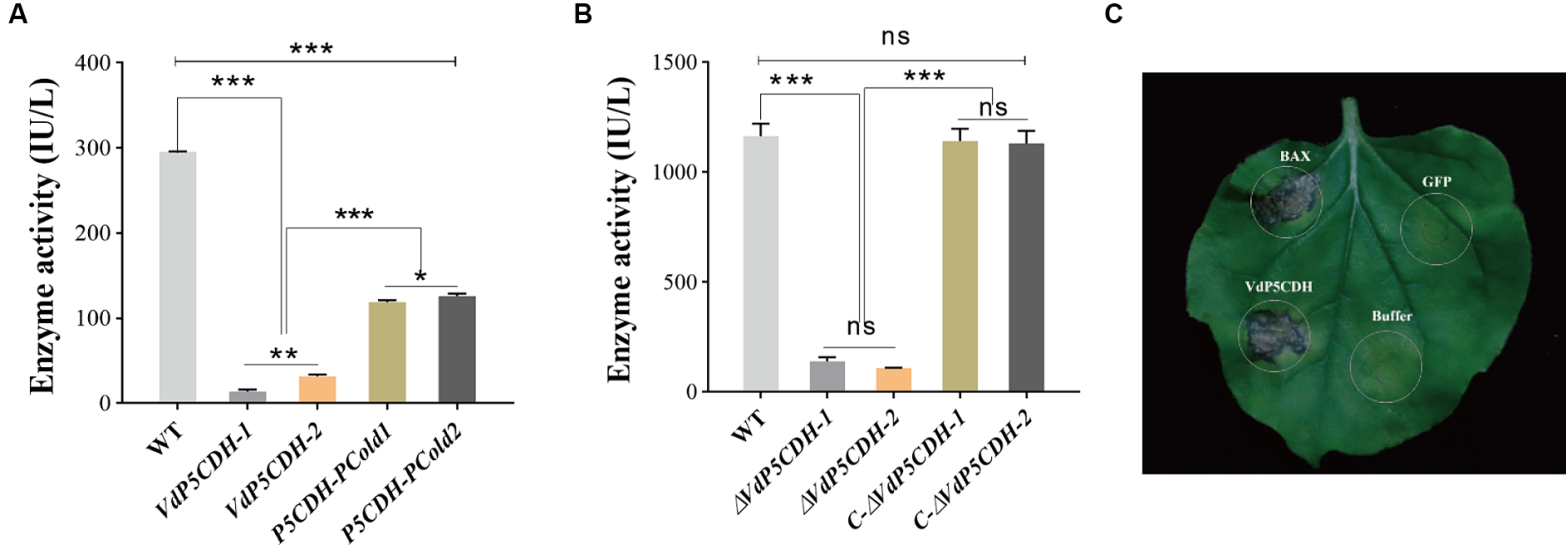
Determination of enzyme activity and the capacity of *VdP5CDH* to induce cell death. **(A,B)** *Carboxylate dehydrogenase* activity in purified proteins **(A)** and conidial suspension **(B)** of the wild-type (WT) strain Vd080, deletion mutans (*ΔVdP5CDH-1* and *ΔVdP5CDH-2*), and complementary mutants (*C-ΔVdP5CDH-1* and *C-ΔVdP5CDH-2*). **(C)** The ability of *VdP5CDH* to induce cell death in *N. benthamiana.* The error bar represents standard error of the mean. *, *p* < 0.05; ***, *p* < 0.001.

The enzyme kinetic analysis revealed that the enzyme activity levels of P5CDH-pCold1 and P5CDH-pCold2 were 107.62 ± 0.80 IU/L and 128.69 ± 0.52 IU/L, respectively (Figure 1A). Furthermore, the dehydrogenase activity in the conidial suspension of the WT strain was measured to be 1163.35 ± 57.02 IU/L, while the enzyme activity levels in the suspension of *ΔVdP5CDH* strains (*ΔVdP5CDH-1* and *ΔVdP5CDH-2*) were significantly reduced to 139.69 ± 18.14 IU/L and 108.29 ± 1.51 IU/L, respectively (Figure 1B). Notably, infiltration with *A. tumefaciens* solution resulted in distinct necrotic spots on *N. benthamiana* leaves after 7 d, indicating that *VdP5CDH* induced characteristic cell death (Figure 1C). The buffer was used as the blank control, BAX as the positive control and GFP as the negative control.

### 3.3 *VdP5CDH* regulated the growth of mycelia and conidia

**Figure 2:**
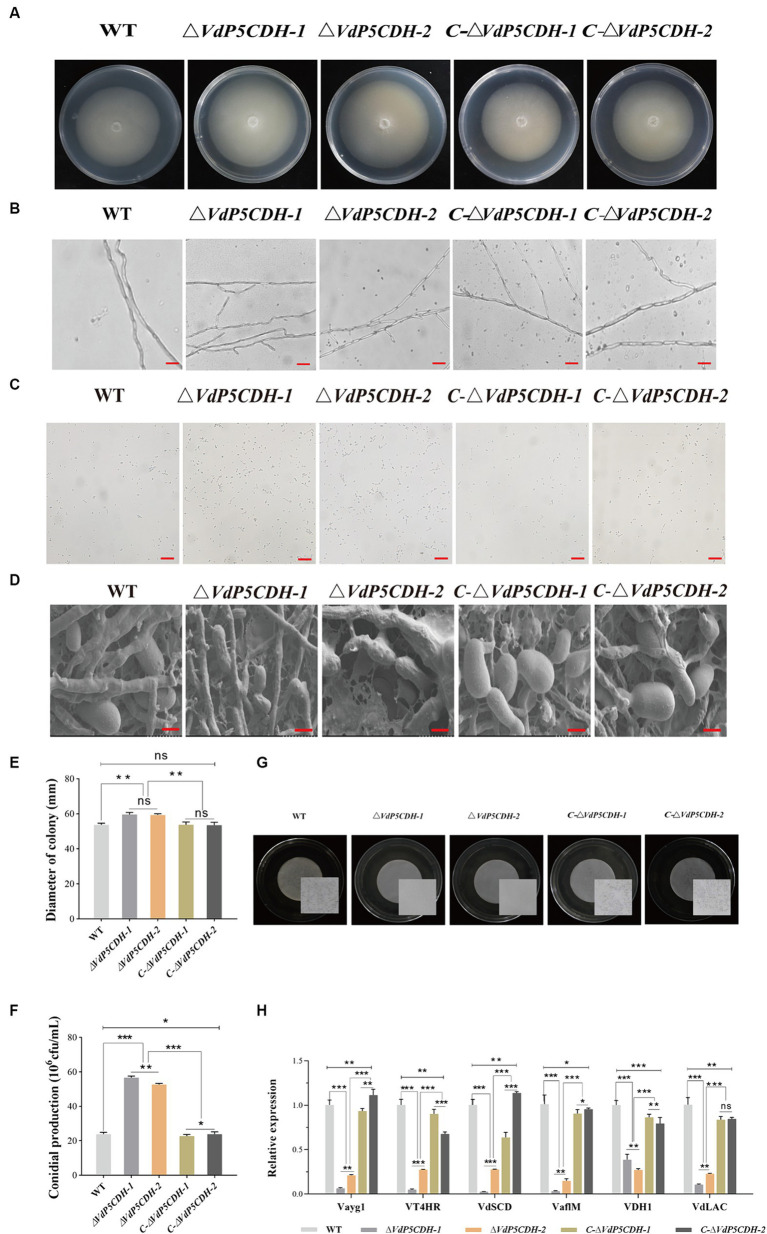
Investigating the role of *VdP5CDH* in the growth of mycelia, conidial production, melanin formation of *V. dahliae*. **(A)** Colony morphology of WT, *ΔVdP5CDH*, and *C-ΔVdP5CDH* strains on PDA medium. **(B)** Morphology of mycelia observed under the optical microscope. Bar, 200 μm. **(C)** Conidial production of all tested strains on Czapek-Dox medium. **(D)** Morphology of conidia from all tested strains examined using scanning electron microscope. Bar, 5 μm. **(E)** Growth diameter of all tested strains shown in panel A. **(F)** The number of conidia of all tested strains as shown in panel C. **(G)** Melanin formation of WT, *ΔVdP5CDH*, and *C-ΔVdP5CDH* strains cultured in BMM medium for 40 d. Microsclerotia magnified view is presented inside the white square using microscopy. Bar, 1 mm. **(H)** The relative expression of melanin-related genes was measured by RT-qPCR. The error bar represents standard error of the mean. *, *p* < 0.05; **, *p* < 0.01; ***, *p* < 0.001.

The radial growth diameter of *ΔVdP5CDH* strain is significantly higher than that of the wild type (Figures 2A,E). Under fluorescence microscopy, the mycelia of WT and *C-ΔVdP5CDH* strains displayed an orderly arrangement without any branching structures, while the mycelia of the *ΔVdP5CDH* strains exhibited increased branching and disordered arrangements (Figure 2B). In addition, the conidial production of the *ΔVdP5CDH* strains were 2.5 folds more than that of the WT and *C-ΔVdP5CDH* strains, and the conidial number per unit time of *ΔVdP5CDH* strains were higher than that of WT and *C-ΔVdP5CDH* strains (Figures 2C,F). Furthermore, the conidia of WT and *C-ΔVdP5CDH* strains appeared plump and smooth, while those of the deletion mutants showed concave surfaces with unevenness, irregular shapes, as well as varying degrees of shrinkage (Figure 2D). Furthermore, *VdP5CDH* regulated melanin production.

The formation of melanin was not observed in the culture medium of the deletion strains. However, a greater amount of denser and darker macroscopic melanin was present on the culture plates of the WT and complementary mutant strains (Figure 2G). The number of microsclerotium reached 316, 260 and 290/cm^2^ for the WT, *ΔVdP5CDH* and *C-ΔVdP5CDH* strains, respectively. Subsequently, the expression of melanin-related genes was analyzed, including *Vayg1*, *VT4HR*, *VdSCD*, *VaflM*, *VDH1* and *VdLAC*. Compared to WT and *C-ΔVdP5CDH* strains, the *ΔVdP5CDH* strains exhibited significantly lower expression levels for these genes (Figure 2H). Therefore, it is evident that *VdP5CDH* played a crucial role in both melanin formation and microsclerotium development.

### 3.4 *VdP5CDH* affects mycelial penetration and morphology

**Figure 3:**
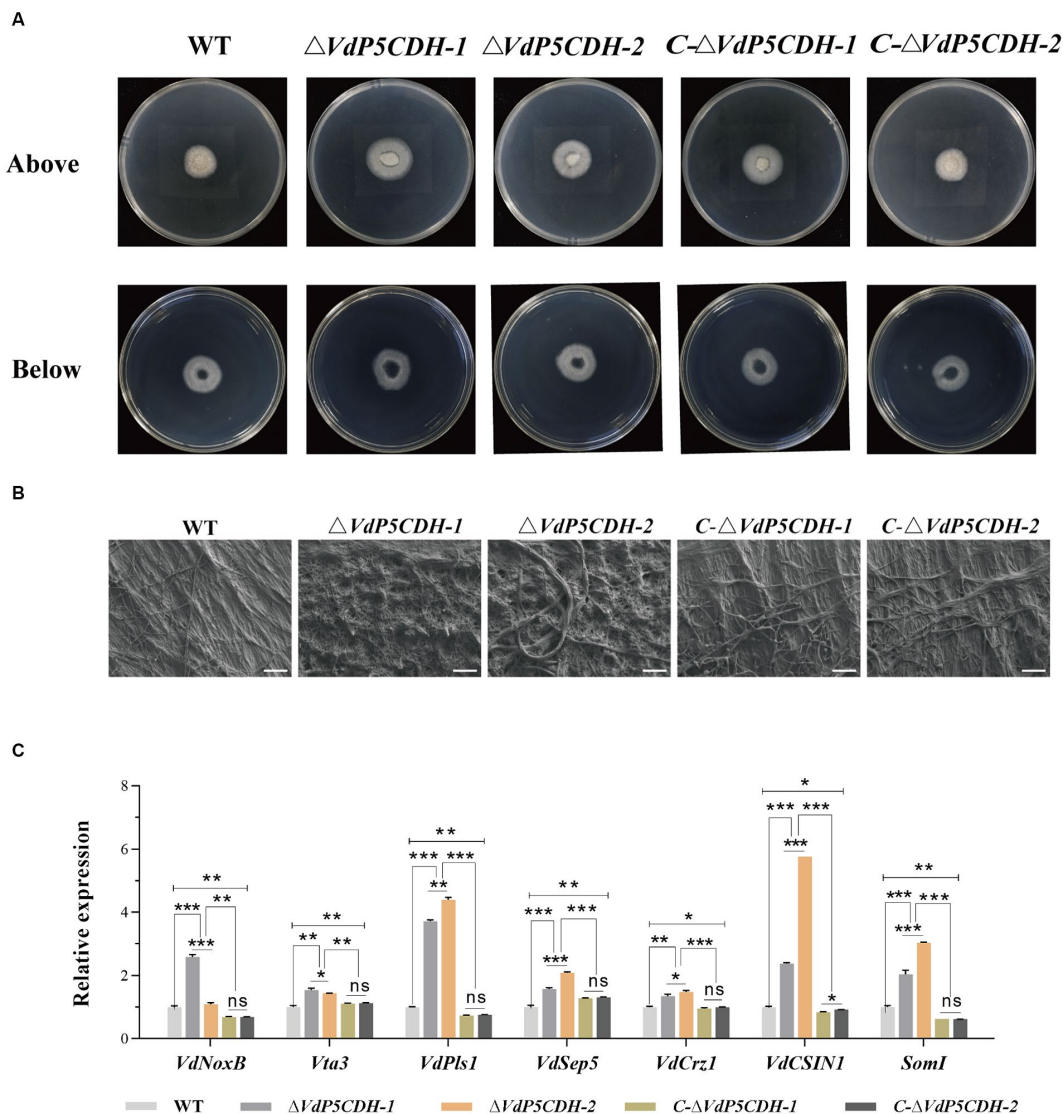
Investigating the role of *VdP5CDH* in mycelium penetration. **(A)** Observation of growth morphology of WT, *ΔVdP5CDH* and *C-ΔVdP5CDH* strains after penetrating cellophane membranes. (**(A)**, Bottom) indicated cellophane was removed for 3 d. **(B)** Micromorphology of mycelium of all tested strains was observed using a scanning electron microscope. Bar, 200 μm. **(C)** RT-qPCR assays were performed to determine the expression of genes associated with infection structure. The error bar represents standard error of the mean. *, *p* < 0.05; **, *p* < 0.01; ***, *p* < 0.001.

The penetration ability of mycelia was assessed using the cellophane penetration test. All strains grew on the PDA medium coated with cellophane for 3 d, and the growth morphology was similar. All strains were able to penetrate cellophane and sustain growth on PDA medium, with *ΔVdP5CDH* displaying enhanced mycelial growth diameter compared to WT and *C-ΔVdP5CDH* strains (Figure 3A). Scanning electron microscopy revealed distinct microscopic morphologies of WT and mutant strains on cellophane. The pattern of fungi is radiant, and the number of branches represents the potential growth ability. The more branches there are, the easier the mycelium is to disorderly distribution. WT and *C-ΔVdP5CDH* strains displayed regular veiny growth patterns, while *ΔVdP5CDH* strains exhibited branching and disorderly mycelial growth, indicating a more vigorous trend (Figure 3B). Moreover, the expression levels of infection peg development related genes *VdNoxB*, *VdPls1*, as well as appressorium structure-related gene *VdCSIN1* were significantly up-regulated in *ΔVdP5CDH* compared to WT and *C-ΔVdP5CDH* strains. Furthermore, in cotton roots, the expression levels of certain genes associated with infection nail development and osmotic colonization, including *Vta3*, *VdSep5*, and *Som1* were also elevated (Figure 3C). Taken together, these results suggested that the deletion of *VdP5CDH* had no influence on the penetration ability of mycelia.

### 3.5 Transcriptome sequencing

**Figure 4:**
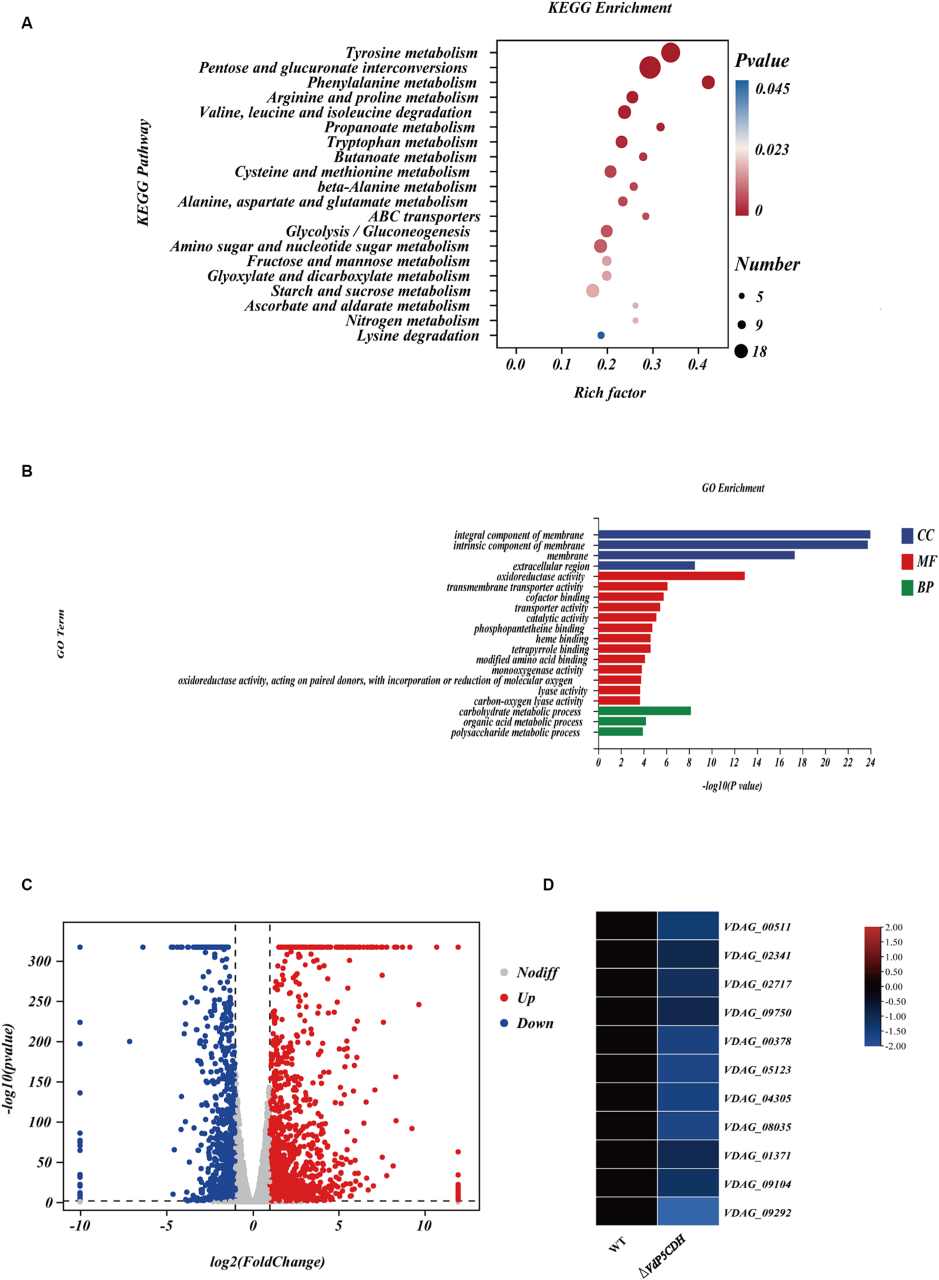
Transcriptome analysis. **(A)** The Kyoto Encyclopedia of Genes and Genomes (KEGG) classification of differentially expressed genes (DEGs). The *p*-value represents the statistical significance between the variables, the number indicates the count of enriched genes. **(B)** The Gene Ontology (GO) classification of DEGs. BP, CC, MF indicate biological processes, cellular components, and molecular function, respectively. **(C)** Volcano plot illustrating the distribution of DEGs. The red point represents the upregulated DEGs with statistical significance, the green represents downregulated DEGs with statistical significance, and gray indicates DEGs without statistical significance. **(D)** Heat maps displaying differential expression patterns of genes. The red color signifies relatively higher expression levels and blue color denotes relatively lower expression levels.

Analysis of the differentially expressed genes (DEGs) collected might provide a deeper understanding of the regulatory changes in *V. dahliae*. A total of 1,605 genes were found to be altered upon gene deletion, with 1,192 genes up-regulated and 413 genes down-regulated (Supplementary Figure S2A). This result clarified the gene expression changes resulting from the deletion of *VdP5CDH* in *V. dahliae*. These DEGs were mapped to 20 KEGG pathways, revealing significant enrichment in carbohydrate metabolism, nitrogen metabolism, ABC transport, and other pathways (Figure 4A). Furthermore, the Gene Ontology (GO) terms ‘oxidoreductase activity’, ‘membrane’, and ‘carbohydrate metabolic process’ exhibited the highest representation in molecular function (MF), cellular component (CC), and biological process (BP), respectively. *VdP5CDH* was mainly related to the above functions and pathways (Figure 4B). The pathways consist of differentially DEGs, with 147, 19, 36, respectively. Volcano plot analysis indicated that down-regulated expression genes constituted only a small fraction of all DEGs identified (Figure 4C). While most genes showed up-regulation in the deletion mutants, a subset of DEGs associated with specific metabolic pathways displayed significant down-regulation as well (Figure 4D; Supplementary Figure S2B). These findings suggest that the deletion of *VdP5CDH* in *V. dahliae* resulted in changes in the expression levels of other genes involved in different pathways with different biological functions.

### 3.6 Utilization of carbon source nutrients by mutants

**Figure 5:**
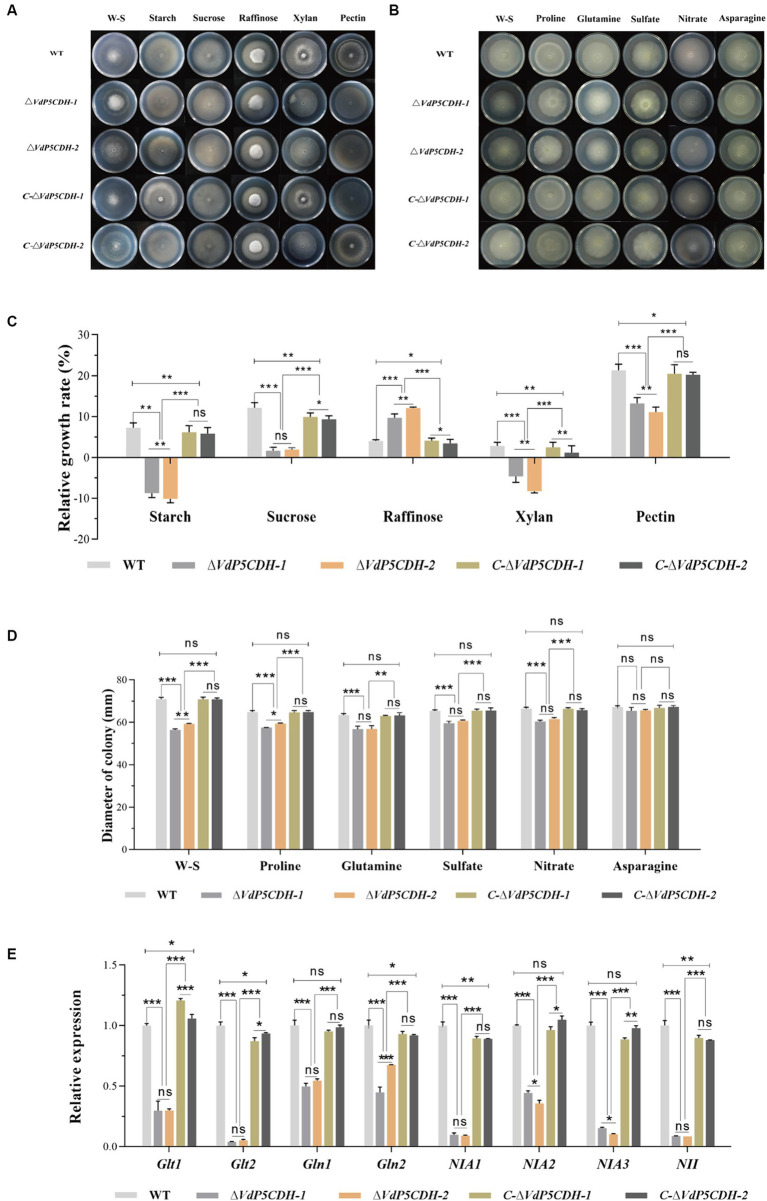
Determination of the role of *VdP5CDH* in utilizing carbon and nitrogen sources. **(A)** Observation of growth morphology of WT, *ΔVdP5CDH* and *C-ΔVdP5CDH* strains on the Czapek-Dox medium supplemented with different carbon sources. **(B)** Observation of growth morphology of WT, *ΔVdP5CDH* and *C-ΔVdP5CDH* strains on the Czapek-Dox medium amended with different nitrogen sources. **(C)** Growth diameter of all tested strains shown in panel A. **(D)** Growth diameter of all tested strains shown in panel B. **(E)** The relative expression of genes related to utilizing nitrogen source was measured by RT-qPCR. Error bar represents standard error of the mean. *, *p* < 0.05; **, *p* < 0.01; ***, *p* < 0.001.

Growth diameters of WT and mutant strains cultured on solid Czapek-Dox medium supplemented with various carbon sources, including starch, sucrose, raffinose, xylan, and pectin, were determined. The results revealed a decrease in the absorption and utilization of sucrose and pectin by *ΔVdP5CDH* strains, which partially hindered their normal growth on the medium. In the medium containing sucrose or pectin as the only carbon source, the mutant strains showed obvious relative growth inhibition. It is worth noting that the mycelia of deletion mutant strains exhibited robust growth on the medium containing raffinose as the carbon source (Figure 5A). Under the culture condition with raffinose or pectin as the only carbon source, the relative growth rate of *ΔVdP5CDH-1* were 7.77 ± 0.98, 10.42 ± 1.39, the relative growth rate of *ΔVdP5CDH-2* were 9.56 ± 0.46, 6.34 ± 1.53, respectively, raffinose and pectin were used as the main carbon sources to promote the growth of deletion strains. Raffinose had a certain promotion effect on the growth of each strain, and the growth rate of WT in the medium containing raffinose was 3.52 ± 0.47. Compared with the *ΔVdP5CDH* strains, the relative growth rate of WT decreased by 54.70 and 63.18%, respectively. Raffinose, as a carbon source nutrient, had a great effect on the growth promotion of deletion mutant (Figure 5C). W-S was used as a control group in experiments exploring mycelia growth status on medium containing carbon sources. These results indicated that *VdP5CDH* affected the uptake and utilization of pectin and raffinose carbon sources. During the growth process of deletion mutant, the promotion effect of most carbon sources decreased.

### 3.7 Utilization of nitrogen source nutrients by strains

The mycelial growth diameters of both WT and mutant strains were measured, revealing a significant reduction in the utilization rate of *ΔVdP5CDH* strains for proline, glutamic acid, sulfate, and nitrate. Conversely, the impact of asparagine on mycelial growth diameters was found to be minimal (Figure 5B). Under the culture conditions with proline, glutamic acid, sulfate, nitrate and asparagine as the only nitrogen sources, the colony growth diameters of WT strains were 64.8.8 ± 1.21 mm, 63.48 ± 1.13 mm, 65.44 ± 0.80 mm, 66.46 ± 1.14 mm, 67.20 ± 1.04 mm. The mycelial growth diameters of *ΔVdP5CDH* strains were 60.45 ± 1.08 mm and 54.51 ± 2.43 mm, respectively, and the growth diameters of *ΔVdP5CDH-1* strains decreased by 11.27, 10.43, 8.91, 9.07 and 2.63%, respectively. The growth diameters of *ΔVdP5CDH-2* strains decreased by 8.29, 10.37, 6.94, 7.07 and 2.25%, respectively, the diameter of mycelia was reduced to different degrees in the medium containing different nitrogen source nutrients (Figure 5D). Quantitative analysis demonstrated a significant down-regulation in the expression of genes associated with nitrogen source utilization and transformation. These results indicated that nitrogen element played an irreplaceable role in pathogen growth and metabolism while highlighting a decreased ability of *ΔVdP5CDH* strains to utilize nitrogen nutrients (Figure 5E).

### 3.8 Resistance of *ΔVdP5CDH* strains to different osmotic stresses

**Figure 6:**
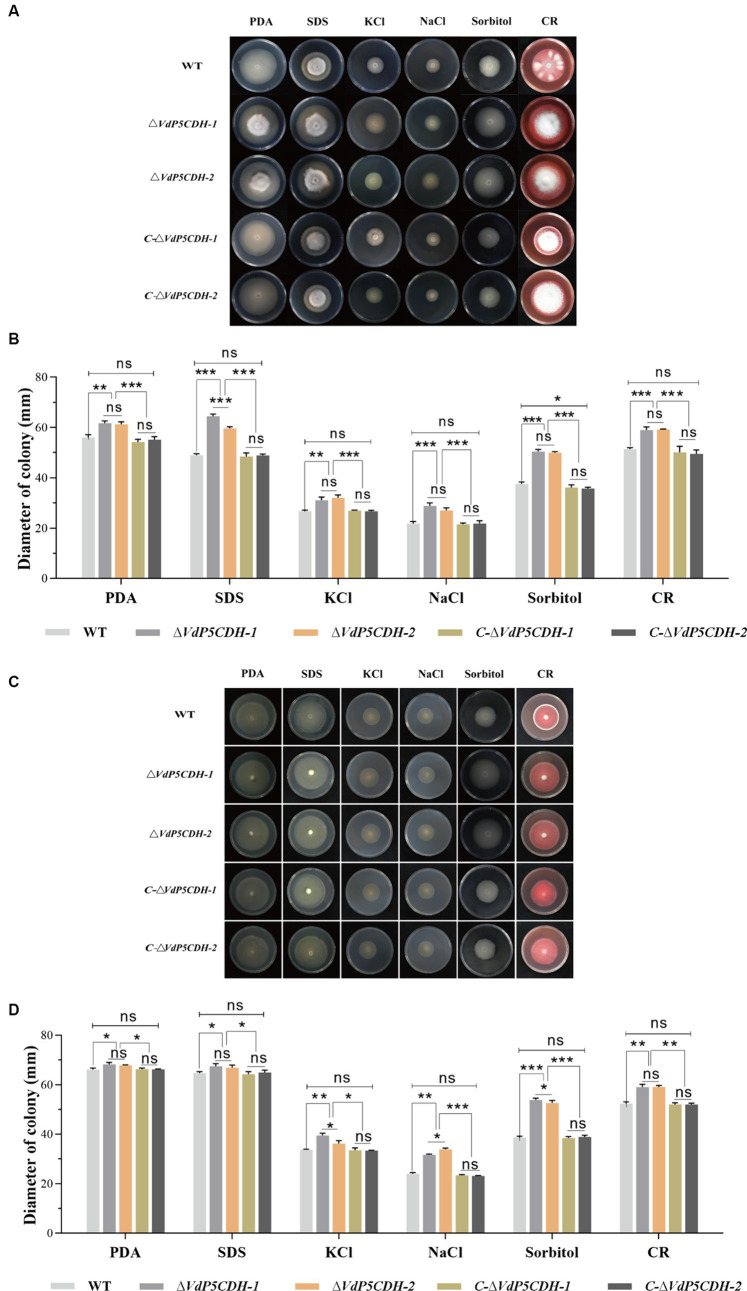
Exploring the role of *VdP5CDH* in resistance to osmotic stress. **(A)** Growth phenotypes of WT, *ΔVdP5CDH*, and *C-ΔVdP5CDH* strains on PDA medium supplemented with 0.002% SDS, 1 M KCl, 1 M NaCl, 1 M sorbitol, and 0.02% Congo red. **(B)** Growth diameter of all tested strains shown in panel A. **(C)** The colony morphology of WT, *ΔVdP5CDH* and *C-ΔVdP5CDH* strains on PDA medium containing glutamic acid and various stress agents. **(D)** Growth diameter of all tested strains shown in panel C. The error bar represents standard error of the mean. *, *p* < 0.05; **, *p* < 0.01; ***, *p* < 0.001.

Under various stress conditions, the growth diameters of *ΔVdP5CDH* strains were significantly larger compared to that of WT and *C-ΔVdP5CDH* strains, especially under SDS, sorbitol and Congo red stress conditions, the stress resistance of *ΔVdP5CDH* strains was significantly enhanced (Figure 6A). On the medium containing SDS, sorbitol and Congo red, the colony growth diameters of WT strains were 49.00 ± 1.05 mm, 37.63 ± 1.35 mm, and 51.39 ± 1.15 mm, respectively. The growth diameters of *C-ΔVdP5CDH-1* strains were 48.49 ± 1.41 mm, 36.16 ± 1.08 mm, 50.17 ± 2.35 mm, respectively. The diameters of *C-ΔVdP5CDH-2* strains were 48.96 ± 0.51 mm, 35.74 ± 0.52 mm, and 49.50 ± 1.58 mm, respectively, indicating that the resistance of WT and *C-ΔVdP5CDH* strains was at the same level under SDS, sorbitol and Congo red osmotic stress. The growth state was the same. The diameters of *ΔVdP5CDH-1* strains were 64.56 ± 0.83 mm, 50.47 ± 0.90 mm, and 58.99 ± 1.25 mm, respectively, which increased by 31.76, 34.12 and 14.79% compared with WT strains, respectively. The diameters of *ΔVdP5CDH-2* strains were 59.63 ± 1.10 mm, 49.96 ± 0.80 mm, and 59.27 ± 0.16 mm, respectively, which increased by 21.69, 32.77 and 15.33%, respectively, compared with WT strains (Figure 6B).

Moreover, when grown on PDA medium supplemented with an additional 10 mM glutamic acid and multiple stressors, the mycelial diameter exhibited a noticeable increase overall (Figure 6C). When 10 mM glutamic acid was added to the medium containing SDS, sorbitol and Congo red stress factors, the growth diameters of *ΔVdP5CDH-1* strains on the medium were 67.44 ± 1.10 mm, 53.81 ± 0.79 mm, and 59.02 ± 1.15 mm, respectively. Compared with WT strains, it increased by 4.23, 39.12 and 12.73%, respectively. The growth diameters of *ΔVdP5CDH-2* strains were 66.85 ± 1.83 mm, 52.61 ± 1.80 mm, and 59.06 ± 1.08 mm, respectively, which increased by 3.32, 36.01 and 12.73% compared with WT strains, respectively. The enhanced response of *ΔVdP5CDH* strains to cell membrane and wall stress was highlighted (Figure 6D).

### 3.9 Assays of the role of *VdP5CDH* in resistance to drug stress, different culture temperature and oxidative stress

**Figure 7:**
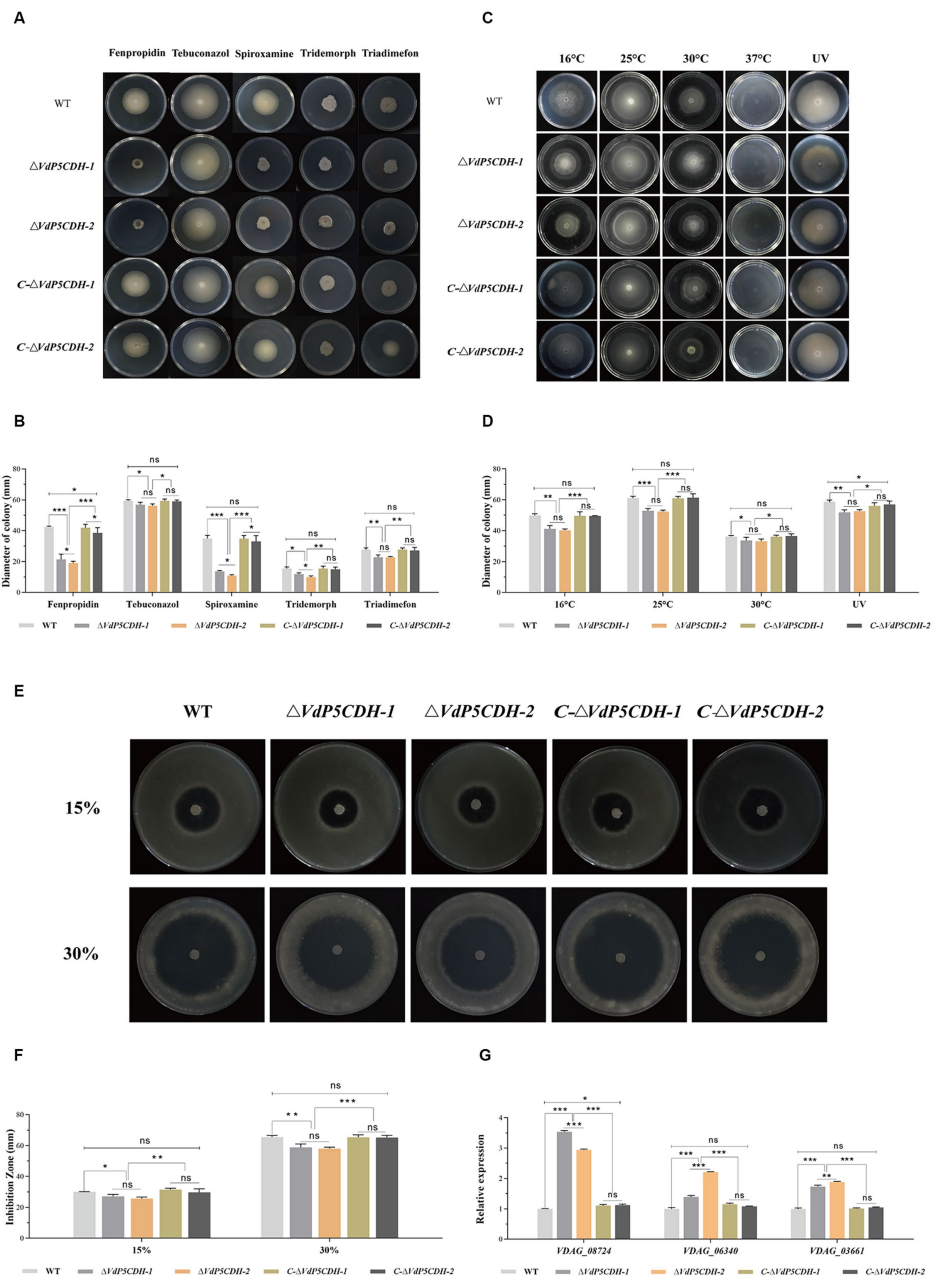
The effects of *VdP5CDH* on drug resistance, temperature variation and oxidative stress were determined. **(A)** Growth phenotypes of WT, *ΔVdP5CDH* and *C-ΔVdP5CDH* strains on PDA medium supplemented with fenpropidin (1 μg/mL), tebuconazol (0.1 μg/mL), spiroxamine (0.5 μg/mL), tridemorph (2 μg/mL), and triadimefon (20 μg/mL). **(B)** Growth diameter of all tested strains shown in panel A. **(C)** Colony morphology of tested strains on PDA medium under different culture temperatures (16°C, 25°C, 30°C, 37°C) or exposure to ultraviolet light for 10 s. **(D)** Growth diameter of all tested strains shown in panel C. **(E)** Observation of the inhibition zone morphology of WT, *ΔVdP5CDH* and *C-ΔVdP5CDH* strains on the medium containing oxidants in different concentrations. **(F)** The diameter of inhibition zone of all tested strains shown in panel E. **(G)** Detecting the relative expression of genes related to oxidative stress by RT-qPCR. The error bar represents standard error of the mean. *, *p* < 0.05; **, *p* < 0.01; ***, *p* < 0.001.

Growth state of the strains varied in response to different stress agents present in the culture environment. Fenpropidin and spiroxamine significantly inhibited the growth of deletion mutant strains, furthermore, compared to WT, *ΔVdP5CDH* strains exhibited a significant reduction in the growth diameter when exposed to the medium containing fenpropidin and spiroxamine (Figure 7A). The growth diameters of WT strains were 42.54 ± 0.57 mm and 34.88 ± 3.61 mm, respectively, in the medium containing fenpropidin and spiroxamine. The growth diameters of *C-ΔVdP5CDH-1* strains were 41.99 ± 2.17 mm and 34.82 ± 2.05 mm, respectively. The diameters of the *C-ΔVdP5CDH-2* strains were 38.59 ± 3.33 mm and 33.09 ± 3.72 mm, respectively. The resistance of the WT and *C-ΔVdP5CDH* strains to the two drugs was little different. The growth diameters of *ΔVdP5CDH-1* strains were 21.44 ± 3.37 mm and 20.48 ± 1.42 mm, respectively, which decreased by 49.60 and 41.28% compared with WT strains, respectively. The diameters of *ΔVdP5CDH-2* strains were 19.13 ± 1.86 mm and 20.56 ± 0.80 mm, respectively, which decreased by 55.03and 41.06% compared with WT strains. It could be seen from this that fenpropidin and spiroxamine have a strong growth inhibition on *ΔVdP5CDH* strains (Figure 7B).

In addition, the mycelial growth of nearly all strains was completely suppressed at a high temperature of 37°C. The growth of *ΔVdP5CDH-1* and *ΔVdP5CDH-2* strains was also significantly inhibited under low temperature conditions (16°C) or short-term ultraviolet irradiation treatment (Figure 7C). In addition, under the conditions of culture temperature at 16°C, or short-term ultraviolet irradiation, the colony growth diameters of WT strains on the medium were 49.89 ± 1.98 mm and 58.80 ± 1.88 mm, respectively. The colony growth diameters of *C-ΔVdP5CDH-1* strains were 49.66 ± 2.70 mm and 56.10 ± 2.01 mm, respectively. The diameters of *C-ΔVdP5CDH-2* strains were 49.61 ± 0.13 mm and 57.09 ± 2.04 mm, respectively, and the growth state of WT and *C-ΔVdP5CDH* strains were roughly the same under the same stress. The growth diameters of *ΔVdP5CDH-1* strains were 41.28 ± 2.06 mm and 51.91 ± 1.58 mm, respectively, which decreased by 17.26 and 11.72% compared with WT strains, respectively. The growth diameters of *ΔVdP5CDH-2* strains were 40.24 ± 1.56 mm and 52.68 ± 1.61 mm, respectively, which decreased by 19.34 and 10.41% compared with WT strains, respectively. It could be seen that the *ΔVdP5CDH* strains were more sensitive to 16°C and short-time ultraviolet irradiation (Figure 7D).

Compared with 15% hydrogen peroxide as the oxidant, the diameter of the inhibition zone increased significantly after adding 10 μL 30% hydrogen peroxide to the center of the medium mixed with conidial filtrate and cultured at 25°C for 5 d. The inhibitory effect of the 30% oxidant stress on mycelial growth was more pronounced (Figure 7E). In addition, on the surface of medium containing 30% hydrogen peroxide, the mycelial growth diameter of the WT strain was 65.52 ± 1.85 mm, while the growth diameters of *C-ΔVdP5CDH-1* and *C-ΔVdP5CDH-2* strains were 65.39 ± 1.63 mm and 65.24 ± 1.45 mm, respectively. There were no significant differences in stress resistance and sensitivity between WT and *C-ΔVdP5CDH* strains. The growth diameters of *ΔVdP5CDH-1* and *ΔVdP5CDH-2* strains were 58.83 ± 2.26 mm and 58.05 ± 1.57 mm, respectively, which decreased by 10.21 and 11.40% compared with WT strains, respectively. The inhibition circle diameters of *ΔVdP5CDH* strains were significantly smaller than that of WT and *C-ΔVdP5CDH* strains, indicating that the deletion of gene could enhance the ability of strains to cope with oxidative stress, and *VdP5CDH* could regulate the sensitivity of strains to antioxidant stress (Figure 7F). Notably, the gene expression related to the antioxidant process was significantly up-regulated in these deletion mutants, suggesting reduced sensitivity, increased antioxidant capacity, and enhanced pathogenicity (Figure 7G).

### 3.10 Regulation of *VdP5CDH* to virulence

**Figure 8:**
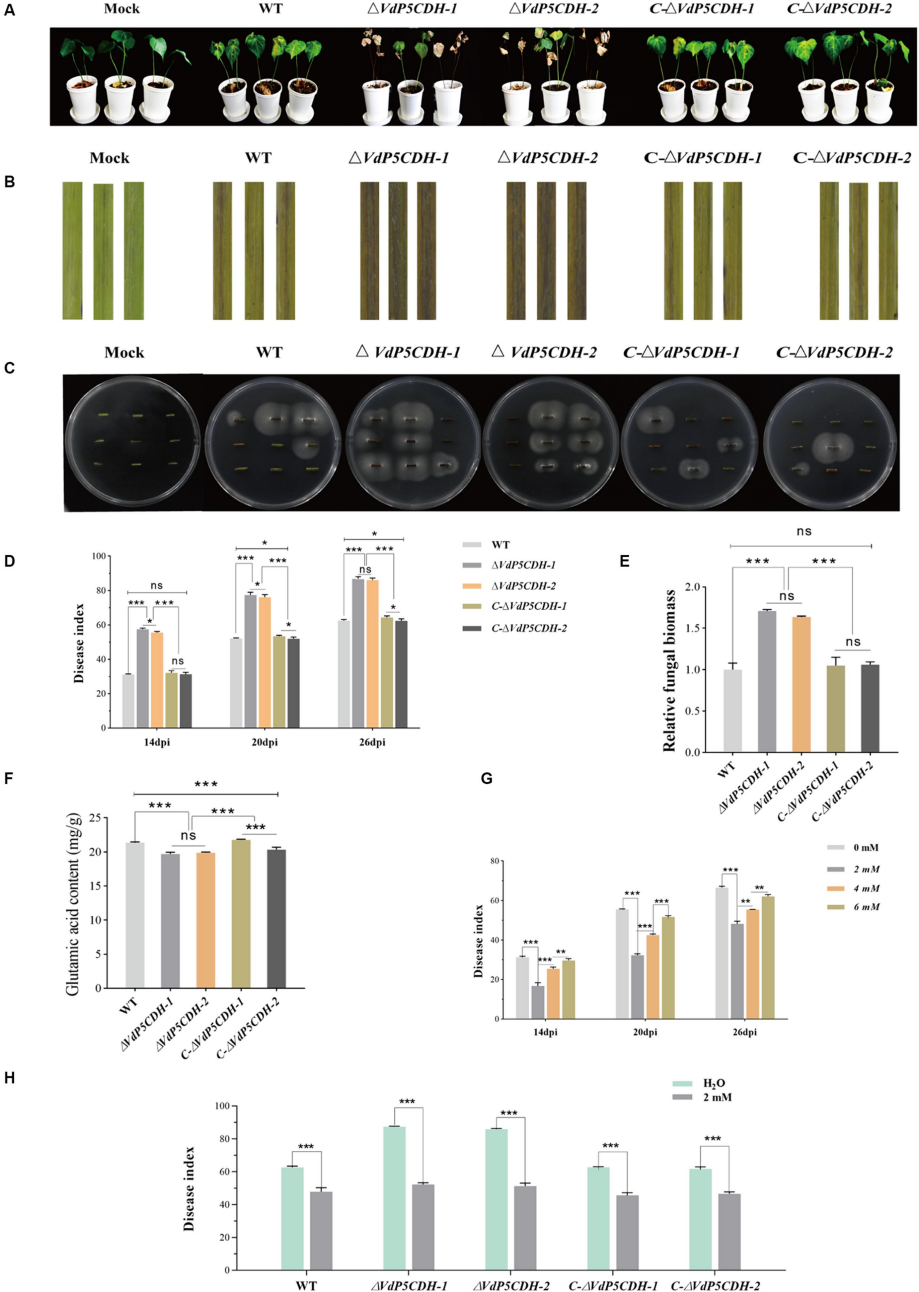
Regulatory effect of *VdP5CDH* and glutamate on pathogenicity **(A)** Growth phenotype of cotton seedlings at 26 dpi of infection by WT, *ΔVdP5CDH* and *C-ΔVdP5CDH* conidial filtrate. **(B)** Brown phenotype of vascular bundle in longitudinal section of cotton stem. **(C)** Experiments on isolation and recovery of pathogenic fungi. **(D)** Statistics of disease index of cotton seedlings. **(E)** Determination of fungal biomass in cotton stems. **(F)** Detection of glutamate content of WT, *ΔVdP5CDH*, and *C-ΔVdP5CDH* strains. **(G)** The disease index of cotton seedlings infected by pathogenic fungi and treated with glutamate was investigated. **(H)** The spraying effects of 2 mM glutamate on pathogenicity of different strains. The error bar represents standard error of the mean. *, *p* < 0.05; **, *p* < 0.01; ***, *p* < 0.001.

In the virulence test on cotton seedlings, at 14 dpi, cotton leaves began to show yellowing and wilting, which gradually worsened with the extension of the growth cycle. At 26 dpi, the cotton plants infected with the *ΔVdP5CDH* strains showed severe leaf chlorosis and browning of vascular bundles in the longitudinal section of the root compared to the plants inoculated with the WT and *C-ΔVdP5CDH* strains (Figures 8A,B). Furthermore, more fungal colonies grew from excised stem sections of seedlings inoculated with the *ΔVdP5CDH* compared to those inoculated with WT and *C-ΔVdP5CDH* strains (Figure 8C). Disease index on plants inoculated with *ΔVdP5CDH* was significantly higher than plants inoculated with the WT and *C-ΔVdP5CDH* (Figure 8D). (Figure 8D). Quantitative analysis of the fungal biomass of cotton stem segment by qPCR revealed that the fungal biomass inoculated with the *ΔVdP5CDH* suspension was approximately 1.7-folds higher than that inoculated with the WT conidial suspension. Therefore, it could be concluded that the infection ability of *V. dahliae* enhanced and the pathogenicity towards cotton increased in the *ΔVdP5CDH* strains (Figure 8E).

### 3.11 Spraying glutamate affected pathogenicity of *Verticillium dahliae*

The glutamate content in WT and mutant strains was quantified using high performance liquid chromatography (HPLC), the contents of *ΔVdP5CDH* strains were 19.70 ± 0.25 mg/g and 19.88 ± 0.11 mg/g, which were also 6.93–7.77% lower than that of WT, the findings revealed a significant reduction in glutamate levels in *ΔVdP5CDH* strains compared to WT (Figure 8F). Cotton seedling roots were inoculated with pathogen solution using the root dipping method, followed by batch application of glutamic acid at varying concentrations. After inoculation with WT conidial suspension for 26 d and spraying with water as a control group, the disease index reached 66.50 ± 1.31; however, the disease index decreased to 48.20 ± 1.35, 55.42 ± 0.07 and 62.10 ± 0.90 after treatment with glutamic acid at concentrations of 2 mM, 4 mM, and 6 mM, respectively. It was observed that the pathogenicity of the pathogens could be significantly reduced when applying a concentration of 2 mM glutamate (Figure 8G). Then another batch of cotton seedlings were inoculated with WT and mutant strains conidial suspensions respectively, and the effects of spraying 2 mM glutamate on cotton seedlings were examined. Spraying water was used as the control. The results demonstrated that after inoculation with WT conidial suspension for 26 d with spraying water, the disease index reached 62.57 ± 1.54, however, the disease index decreased to 47.81 ± 2.42 after spraying with 2 mM glutamic acid, which decreased by 23.59%. Similarly, after inoculation with *ΔVdP5CDH-1* and *ΔVdP5CDH-2* conidial suspensions for 26 d with spraying water, the disease indexes were recorded as 87.34 ± 0.60 and 85.85 ± 0.71, respectively, while the disease indexes of cotton seedling inoculated with *ΔVdP5CDH* suspensions and sprayed with 2 mM glutamic acid decreased to 52.19 ± 1.03 and 51.27 ± 1.76, which decreased by 40.25 and 40.28% compared with the control group, respectively (Figure 8H). The results indicated that the application of 2 mM glutamate could significantly reduce the virulence of *ΔVdP5CDH* strains than that of WT.

## 4 Discussion

The enzyme activity assay is conducted at 50°C and a pH range of 6 to 10, utilizing Δ1-pyrroline-5-carboxylate as the substrate, indicating a significant reduction in carboxylate dehydrogenase activity in the deletion mutants. Two primary categories of unconventional secretion pathways (UPS) are recognized: (I) the extracellular secretion of cytoplasmic proteins lacking signaling peptides and (II) the transport of transmembrane proteins with signaling peptides directly to the cell surface (Golgi-bypassing) (Kim et al., 2018). UPS mechanisms exist in various organisms, including yeast, fungi, plants, drosophila, and mammals (Rabouille and Malhotra, 2012). This study demonstrated that the protein encoded by *VdP5CDH* may exhibit an unconventional secretory pathway as it triggers a hypersensitive response (HR) in the absence of signaling peptides (Figure 1C).

The proliferation of mycelia and conidia plays a crucial role in the pathogenesis of fungi, as evidenced by extensive research (Luo et al., 2014; Xiong et al., 2016). *VDH1* encodes a hydrophobic protein that regulates the mycelia development and microsclerotium formation (Klimes and Dobinson, 2006; Klimes et al., 2008). Deletion mutants of *VdNLP1* and *VdNLP2* have been observed to promote vigorous growth of aerial mycelium (Zhou et al., 2012). *VdGRP1* inhibits mycelial growth and facilitates the formation of microsclerotia (Gao et al., 2010). The studies on the microsclerotia of *V. dahliae* revealed that the gene deletion mutants play a crucial role in regulating the development, maturation of microsclerotia, and the accumulation of melanin. During the formation and maturation of microsclerotium, the production of 1,8-dihydroxynaphthalene (DHN)-melanin by pathogenic fungi significantly contributes to stress resistance (Luo et al., 2019; Eisenman et al., 2020). Notably, the common negative transcription regulatory factors *VdPKS9* and *VdMRTF* in *V. dahliae* play a regulatory role in melanin biosynthesis, micronucleus formation and virulence (Lai et al., 2022; Li et al., 2022). *VdCrz1* plays an extraordinary role in pathogen signal transduction by maintaining the integrity of cell wall, promoting infective peg, microsclerotium development, and enhancing virulence (Xiong et al., 2015). Absence of *VdP5CDH* enhanced conidia germination, accompanied by crumpled and depressed microscopic morphology (Figures 2C,D,F). Although *ΔVdP5CDH* mutants exhibited disorganized microscopic mycelial status and no melanin production, their competency to penetrate cellophane was unaffected (Figures 2G, 3A). Fungal infections are commonly associated with the degradation of the host cell wall (Lorrai and Ferrari, 2021), where enzymes target easily degradable cell wall components such as hemicellulose and pectin, while more resistant secondary cell walls composed mainly of xylan, cellulose, and lignin form on the inner surface (Xiao et al., 2021). The absence of A-oxoglutarate dehydrogenase VdOGDH was found to restrict the growth of mutant strains on media containing only sucrose, pectin, xylan, and galactose as carbon sources, indicating that VdOGDH plays a role in carbon nutrient utilization by the pathogen (Li et al., 2020). The supply of nitrogen nutrients and the activation of related downstream metabolic pathways are conducive to promoting adaptive survival, virulence and the energy acquisition required for growth and colonization (Schure et al., 2000). *ΔVdP5CDH* mutants significantly promoted mycelial growth when raffinose and pectin were utilized as carbon nutrients and a decreasing trend in the growth rate of multiple nitrogen sources (Figures 5C,D).

*VdSsk1* and *VdSsk2* participate in the response mechanism against various abiotic stresses, and gene deletion renders strains more susceptible to NaCl and sorbitol (Zheng et al., 2019). Previous research has established that certain genes are instrumental in regulating the growth and developmental processes of pathogenic fungi, as well as in enhancing their resistance and tolerance to stress (Zhang et al., 2022). In present study, the application of fungicides remains an effective approach for preventing and controlling *V. dahliae* infections. Deletion mutants lacking *Crz1* exhibit increased sensitivity to fluconazole. The enzyme HsP5CDH has been identified as a direct target, and its overexpression has been shown to counteract the accumulation of reactive oxygen species (ROS) induced by H_2_O_2_ or UV exposure (Yoon et al., 2004). The concurrent induction of ProDH and P5CDH under low temperature stress supports the hypothesis that P5C or glutamyl semi-aldehyde (GSA) accumulation is inhibited under such conditions. Substrate channels have been reported in bacterial proline catabolism (Sanyal et al., 2015), as well as proline dehydrogenase TtProDH and carboxylate dehydrogenase TtP5CDH are certified in mono-functional thermophile bacteria (Srivastava et al., 2010; Singh et al., 2014). The absence of *VdThit* diminishes pathogen tolerance to UV stress, emphasizing the importance of specific proteins in stress resistance (Zhang et al., 2011). Reactive oxygen species can act as secondary messengers to regulate various biochemical processes at the transcriptional level (Rhee, 2006; D'Autréaux and Toledano, 2007; Wei et al., 2011; Zhang et al., 2015). ProDH facilitates the oxidation of proline to P5C, providing energy for cellular activities and promoting ROS production. Conversely, P5CDH converts P5C to glutamate (Glu), effectively removing it from the a forementioned cycle to reduce ROS production. *ΔVdP5CDH* strains exhibited an increased responsiveness to stresses impacting the cell membrane and cell wall (Figure 6B). In addition, after treatment with various fungicides, low temperature (16°C) or short-term ultraviolet irradiation, the growth of mycelia was significantly inhibited, especially for the deletion mutants (Figures 7B–F).

The antioxidant capacity of mutant strains is also significantly enhanced. In a course of experimentation that involved the application of a concoction of glutamic acid and phosphoric acid onto potato foliage, there were an increment in foliar nitrogen content, alongside a decrement in carbohydrate concentration (Xu et al., 2014). Previous investigations have shown that the exogenous application of glutamic acid and a variety of other amino acids can stimulate the plant growth process (Xu et al., 2017; Zhang et al., 2017). It is also noted that the response to amino acid applications varies across different crop species (Qiao et al., 2017). The results of this study demonstrated a pronounced increase in the pathogenicity of the *ΔVdP5CDH* strains, evidenced by increased fungal colonization. External spraying of 2 mM glutamate mitigated the symptom severity (Figure 8H). *VdP5CDH* played an important role in regulating mycelial morphology, melanin synthesis, stress resistance, and the virulence of fungus, the deletion of gene weakened stress resistance of pathogens and reduced the utilization of nutrients by the strains, indicating that the deletion mutants weakened its own metabolic function and physiological characteristics and increased the production of toxins to survive in response to adverse living conditions.

## 5 Conclusion

In summary, *VdP5CDH* regulated mycelial and conidial growth, melanin formation, nutrient utilization, stress resistance. Additionally, it negatively modulated the virulence of *V. dahliae*. These findings provided a theoretical basis for devising strategies to control plant fungal diseases. *VdP5CDH* regulated mycelial and conidial growth, melanin formation, nutrient utilization, stress resistance. Additionally, it negatively modulated the virulence of *V. dahliae*.

## Data availability statement

The raw data supporting the conclusions of this article will be made available by the authors, without undue reservation.

## Author contributions

WS: Writing – original draft. LZ: Writing – original draft. JZ: Writing – review & editing. HF: Writing – review & editing. YZ: Writing – review & editing. ZF: Writing – review & editing. HZ: Writing – review & editing. FW: Writing – review & editing.
