# Supplementary material for: VdP5CDH is involved in melanin formation, stress resistance and play a regulatory role in virulence of Verticillium dahliae
Source: Front Microbiol. 2024 Jul 24;15:1429755. doi: 10.3389/fmicb.2024.1429755 (PMC11303183; doi:10.3389/fmicb.2024.1429755)
Supplement: Supplementary file 1 [file Table_1.DOCX]

**Supplementary material**

**Table S1.** Differential genes associated with virulence differentiation.

| Locus | Gene Name | Description |
| --- | --- | --- |
| VDAG_03714 | sugar transporter | Carbohydrate transport, transmembrane transport |
| VDAG_03218 | delta-1-pyroline-5-carboxylate dehydrogenase | MAPK signal transduction pathways |
| VDAG_06462 | putative substrate translocation pore | MFS superfamilies, transmembrane transport |
| VDAG_04757 | alpha-galactosidase | Glycoside hydrolase 27 family |
| VDAG_10439 | histidinol-phosphatase | Histamine phosphatase activity |
| VDAG 08487 | ubiquitin-conjugating enzyme | Ubiquitin-conjugating enzyme activity |

**Table S2.** Primers used in this study.

| Primer name | Sequence (5’-3’) |
| --- | --- |
| *B303-VdP5CDH*-UP-F | GGACCGGACGGGGCGGTACCCCACGACACGGACGAGACGC |
| *B303-VdP5CDH*-UP-R | CTTCAATATCAGTTAACGTCGGCGGGCAACGATCGCTGCT |
| *B303-VdP5CDH*-Hyg-F | AGCAGCGATCGTTGCCCGCCGACGTTAACTGATATTGAAG |
| *B303-VdP5CDH*-Hyg-R | TTTTCGACGTGGCCCCGACTCTATTCCTTTGCCCTCGGACG |
| *B303-VdP5CDH*-Down-F | CGTCCGAGGGCAAAGGAATAGAGTCGGGGCCACGTCGAAAA |
| *B303-VdP5CDH*-Down-R | TAGTCCCGGGTCTTAATTAAAACGACAGCTTGGTCGGATA |
| *pSULPH-VdP5CDH*(+UP)-F | ACGGCCAGTGCCAAGCTTCCACGACACGGACGAGACGC |
| *pSULPH-VdP5CDH*(+UP)-R | GACGTTCTCGGAGGAGGCCATCGCTTCGTTGCTGGGGTATTC |
| Test-Hyg-F | TCGTTATGTTTATCGGCACT |
| Test-Hyg-R | TCGGTCGGCATCTACTCT |
| *Test-VdP5CDH-526F* | ATTGGAGGCAAAGAGGTAAGG |
| Test-*VdP5CDH*-526R | CAATGGCAGTGAAGTTGAAGG |
| Test-*VdP5CDH*-674F | GCGTCCAACTGGCTCCTCT |
| Test-*VdP5CDH*-674R | TTGCCGCTGTCGTCATACA |
| P5CDH-pCold-F | CCACTTTCAACGAGCTGATGATGAACGCTCGTCGCGCGGCC |
| P5CDH-pCold-R | GGCAGGGATCTTAGATTCTGCTACGCTTCGTTGCTGGGGTA |
| pGR107-*VdP5CDH*-F | GGTCAGCACCAGCTAGCATCGATATGAACGCTCGTCGCGCGGCC |
| pGR107-*VdP5CDH*-R | TCGCCCTTGCTCACCATCCCGGCTACGCTTCGTTGCTGGGGTA |
| *Vayg1*-F | GTTGCGACGAGTTCTTGT |
| *Vayg1*-R | ACCATCACCTTGCCCATA |
| *VT4HR*-F | TGGTGGCATCAAGACAGACA |
| *VT4HR*-R | CGAAGCGAGGAAGCAAACAA |
| *VdSCD*-F | ATGCCCGCTTCCGAGTTC |
| *VdSCD*-R | TTCCACACGCCGTCAATCTT |
| *VaflM*-F | GACTGTCAATGCCATCGCC |
| *VaflM*-R | CGGTGACCTTGATAACTT |
| *VDH1*-F | GTCTATTCATCTGGTTCCTCCCTA |
| *VDH1*-R | CAAACCTCTTACAATGTTGACGC |

**Table S2. (continued)**

| Primer name | Sequence (5’-3’) |
| --- | --- |
| *VdLAC*-F | CGTTTCCTCACTTTAGCCACAGC |
| *VdLAC*-R | CACCCAGTCCACCGTCCATTTGT |
| *VdNoxB*-F | TGCGTGGCAAGCATAAGACATAC |
| *VdNoxB*-R | GACAGCACGAGTGAAATCACCAAC |
| Vta3-F | GATGTCTGCCCTGCGTAA |
| Vta3-R | GATCTGAGCCTGGTCAAAGT |
| *VdPls1*-F | ATGGTCAACAAGATCCTCGCGA |
| *VdPls1*-R | TCCGGCTGCTCAAACATGTTGT |
| *VdSep5*-F | AGCTCGACCTGGACGAGGA |
| *VdSep5*-R | GAGGCTTCGTTATCAATCTCGTCTC |
| *VdCrz1*-F | ATGGATCAGCAAGCTCAACATCG |
| *VdCrz1*-R | GATCCAGACCGAGACCGAGAC |
| *VdCSIN1*-F | CTTTGATTGTGGTATGGGTTCT |
| *VdCSIN1*-R | GTGGTGGGTTTGCCTTGT |
| Som1-F | GTCGTGACAACCGAAAGCAG |
| Som1-R | TCCCTCGTGGAGCGCAAA |
| *VDAG_00511*-F | CCGCCGTACCATTGAAGAGT |
| *VDAG_00511-*R | TCCCAAGCATTCCAGGTGTC |
| *VDAG_02341-*F | ATTTCGTGCTCGGTCACACT |
| *VDAG_02341-*R | CATCGAGTAGATGGGTGGGC |
| *VDAG_02717-*F | CCAACCCAAGCCTGCATTTC |
| *VDAG_02717-*R | TAGCTTTCGGCCCACGATTT |
| *VDAG_09750-*F | TGGAAACGAAGGTGATCGCA |
| *VDAG_09750-*R | ATCGGCGATAGAGTTGCCAG |
| *VDAG_00378-*F | ACCATCAAGGAAGACCGCAG |
| *VDAG_00378-*R | CTTGGTTGACTGTTGGCGTG |
| *VDAG_05123-*F | ACACCGAGGCTGACATTCTG |
| *VDAG_05123-*R | TCCCTTCCATTCATCGCTCG |

**Table S2. (continued)**

| Primer name | Sequence (5’-3’) |
| --- | --- |
| *VDAG_04305-*F | TTCAGCTGGCTCGGTTTCAT |
| *VDAG_04305-*R | CAAACTTGTCACATGCCGGG |
| *VDAG_08035-*F | ATCCCTCGGTACACAGACGA |
| *VDAG_08035-*R | GAGGAAGACAGGCTGCAGTT |
| *VDAG_01371-*F | ATGCTCTAGAGATGCACGGC |
| *VDAG_01371-*R | GACGACCTCACCTGACGTTT |
| *VDAG_09104-*F | ACCAGTCCTCAGGAGCGTAT |
| *VDAG_09104-*R | CTCTTGACACGCTGACCCTT |
| *VDAG_09292-*F | ACGTTGGTTGTCCCTACGAC |
| *VDAG_09292-*R | TGTGTATTGGTGCAGTCGCT |
| *Glt1*-F | CCGAGGAGAACATCATCATT |
| *Glt1*-R | TAGGCGATACCACCAGAC |
| *Glt2*-F | CAAGCCTAGCCACAAGATT |
| *Glt2*-R | TGTCTTCCAACTGCCTCT |
| *Gln1*-F | ACTTGCCGAGAAGGAGTA |
| *Gln1*-R | TCATGGCGGTAGTTGAAC |
| *Gln2*-F | TACACGCCTGATGACCTT |
| *Gln2*-R | GTGTACTCCTGCTCAAGG |
| *NIA1*-F | CGCTCGCTATGTATCCTT |
| *NIA1*-R | GTTATCGCTTGGCTCCTT |
| *NIA2*-F | ACAAGACCGAGAAGGACAT |
| *NIA2*-R | GTGCTCGTTGTGGTACTT |
| *NIA3*-F | CGAGGAGGTCATGCTTGA |
| *NIA3*-R | AGAGGTGTCGGCGTTGTT |
| *NII*-F | TGGATGCCGACAACTATG |
| *NII*-R | CAACACCAGGAGCGATTA |
| *VDAG_08724-F* | GAGCCGACCGAAAGCGAGAG |
| *VDAG_08724-R* | AGCCATGACACCCAAGCCAAG |

**Table S2. (continued)**

| Primer name | Sequence (5’-3’) |
| --- | --- |
| *VDAG_06340*-F | TTCCTCGCTGATGCCGTGAAG |
| *VDAG_06340*-R | TTGCGTGTAGCCTTCCTCCTTG |
| *VDAG_03661-F* | ACTACGGCGTCATCACACTCAAG |
| *VDAG_03661-R* | TCCAGCACCATCCTTCGCAATC |
| *β-tubulin*-F | TCACCAGCCGTGGCAAGGTTG |
| *β-tubulin*-R | AGCAAAGGGCGGTCTGGACGTTG |
| *GhUBQ7*-F | GAAGGCATTCCACCTGACCAAC |
| *GhUBQ7*-R | CTTGACCTTCTTCTTCTTGTGCTTG |
