# Supplementary figures and images for: VdP5CDH is involved in melanin formation, stress resistance and play a regulatory role in virulence of Verticillium dahliae
Source: Front Microbiol. 2024 Jul 24;15:1429755. doi: 10.3389/fmicb.2024.1429755 (PMC11303183; doi:10.3389/fmicb.2024.1429755)

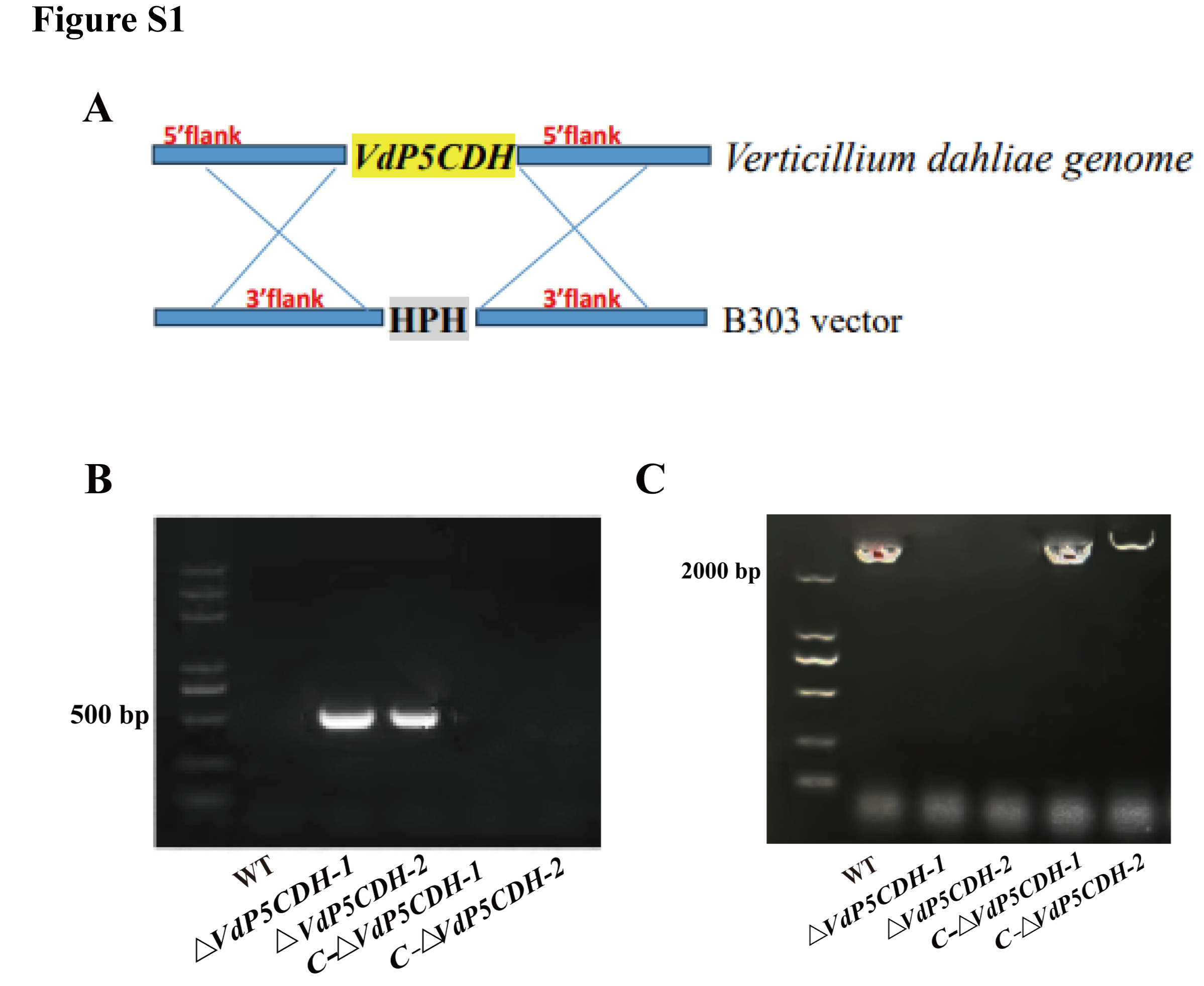

Supplement: Supplementary file 2 [file Image_1.TIF]

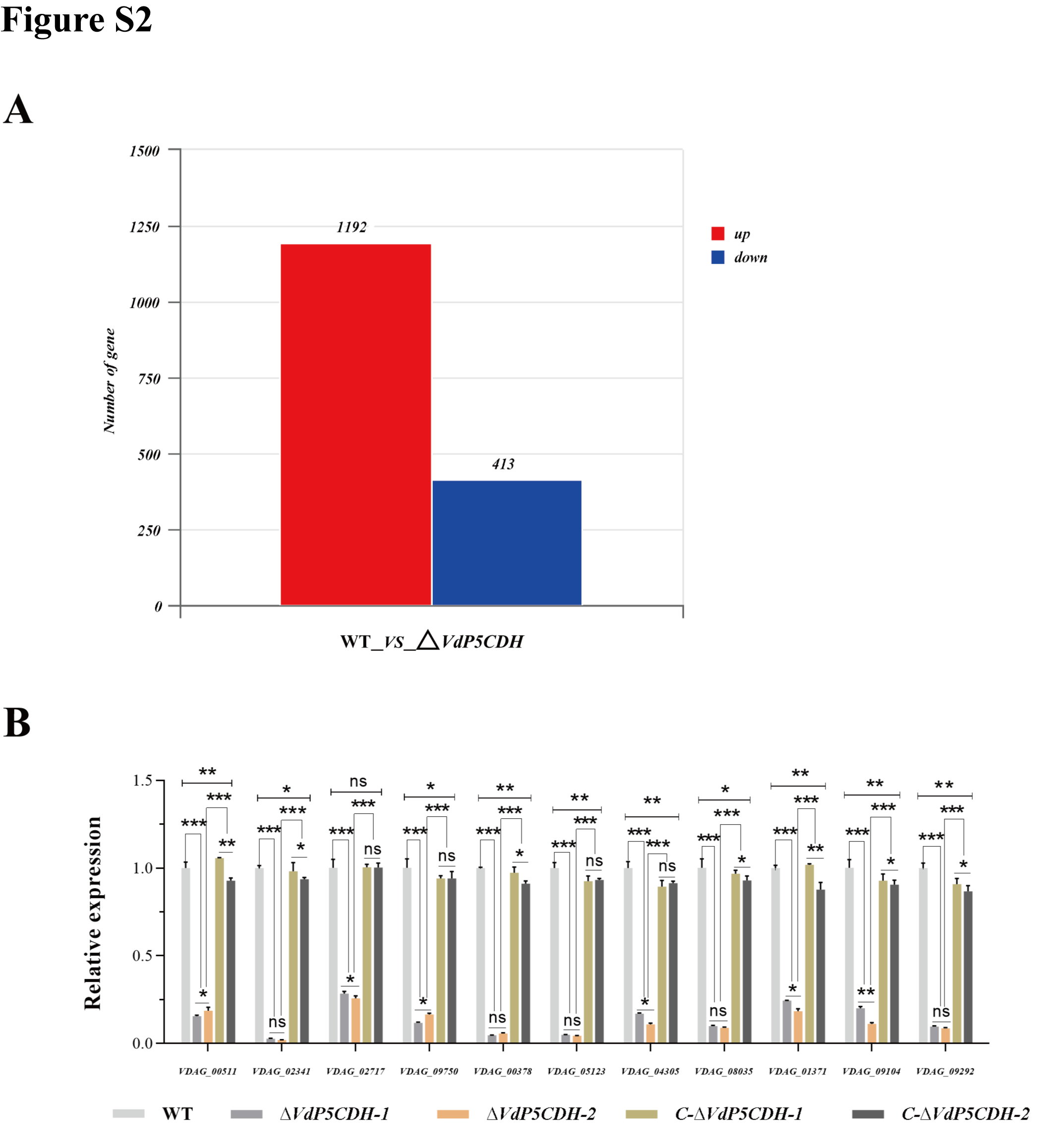

Supplement: Supplementary file 3 [file Image_2.TIF]
